# Diagnostic performance of optical spectral transmission compared to magnetic resonance imaging in patients with inflammatory arthritis

**DOI:** 10.1186/s13075-025-03478-y

**Published:** 2025-01-30

**Authors:** Konstantinos Triantafyllias, Mohammed Alhaddad, Andreas Schwarting, Veronika Balaklytska, Xenofon Baraliakos

**Affiliations:** 1 Department of Rheumatology, Acute Rheumatology Centre Rhineland-Palatinate, Kaiser-Wilhelm-Str. 9-11, 55543 Bad Kreuznach, Germany; 2 Department of Internal Medicine I, Division of Rheumatology and Clinical Immunology, Johannes Gutenberg University Medical Centre, Mainz, Germany; 3 Johannes Gutenberg University Mainz, Mainz, Germany; 4 Rheumazentrum Ruhrgebiet Herne, Ruhr University Bochum, Herne, Germany

**Keywords:** Optical spectral transmission, Magnetic resonance imaging, RAMRIS, Inflammatory arthritis, Disease activity

## Abstract

**Background:**

Optical spectral transmission (OST) is a modern diagnostic method capable of quantifying inflammation in the finger and wrist joints of arthritis patients by assessing the blood-specific absorption of light transmitted through a tissue. The diagnostic performance of this modality has not been adequately examined and data regarding OST associations with magnetic resonance imaging (MRI) are limited. Aim of this study was therefore to investigate the performance of OST in assessing joint inflammation as compared to MRI in patients with inflammatory arthritis (IA).

**Methods:**

Data from patients who underwent MRI and OST for suspected IA were analyzed. For comparison, a historical healthy control (HC) group with OST was also accounted. MRI findings were quantified using the Rheumatoid Arthritis MRI Score (RAMRIS). Diagnostic accuracy of OST was evaluated using Receiver Operating Characteristics (ROC), while correlation analyses were conducted to explore relationships between OST and MRI, as well as disease activity markers.

**Results:**

Overall, *71* patients with known rheumatic diseases (*n* = 1,*542* wrist and finger joints) and *114* HC (n = *2*,*508* joints) subjects were included. *51* patients showed inflammatory signs on MRI (MRI+). These also showed significantly higher OST scores (*16.41 ± 5.53)* than subjects without MRI inflammation (MRI-) (*11.52 ± 5.03*) or HC *(10.78 ± 4.19) (all;* p < *0.001*). OST showed significant correlations with RAMRIS-synovitis and tenosynovitis scores in the MRI + group (rho = *0.541*, p < *0.001;* rho = *0.341*, p = *0.01*, respectively). Significant correlations were observed between OST and clinical parameters for disease activity. Using MRI as a reference, the best diagnostic value of OST was observed at the wrist level in the MRI + group, by an AUC of 0.*833* (*95*%CI *0.700-0.966*).

**Conclusion:**

OST showed an excellent performance compared to MRI and correlated significantly with RAMRIS scores and clinical parameters in IA patients, also differentiating IA from HC.

**Supplementary Information:**

The online version contains supplementary material available at 10.1186/s13075-025-03478-y.

## Introduction

In recent years, it has become evident that precise monitoring of disease activity in individuals with inflammatory arthritis using tight control strategies is a critical factor for effectively controlling inflammation, preventing disease progression, and ultimately improving overall outcomes [1–4]. However, implementing such management approaches can pose challenges. Chief among these challenges is the difficulty in adhering to tight control strategies, often due to time constraints and inadequate rheumatologist resources [5, 6]. Furthermore, clinical scores like the Disease Activity Score 28 (DAS28) have limitations, such as examiner dependence and inadequate assessment of subclinical disease activity [7, 8]. As a result, additional diagnostic modalities like MRI and joint ultrasound (US) are often utilized in everyday clinical practice. The advantage of MRI over US is its ability to visualize bone marrow edema in addition to its exceptional visualization of joints and surrounding soft tissues. It is, nonetheless, accompanied by a substantial cost, longer examination times, and limited accessibility [9, 10]. Additionally, arthritis activity assessment via MRI often requires the use of contrast agents. US, on the other hand, is more broadly feasible than MRI; however, it relies on the operator’s expertise and may also be time-consuming, especially in the case of multiple joint examinations and thorough scoring [11–13]. During the last years, alternative imaging tools like optical spectral transmission (OST) (HandScan^®^) [14–19] and fluorescence optical imaging (FOI) (Xiralite^®^) [20–22] have been introduced for the assessment of disease activity in patients with inflammatory arthritides.

In particular, OST offers the potential for evaluating joint inflammation using red and near-infrared light technology without the need for contrast agents or radiation, in contrast to FOI which requires the intravenous injection of indocyanine green with potential risks like allergic reactions [23]. OST can assess the blood-specific absorption of light transmitted through a tissue, thus allowing for a non-invasive quantification of inflammation-associated changes in blood flow [24]. In the case of inflammatory arthritis, the speed and magnitude of blood pooling in the joint increases, due to changes in vascularity [25, 26], resulting in decreased light transmission through the inflamed joints. OST can also be easily assessed by trained medical assistants and nursing staff. Moreover, the quantification of OST results is automated by the OST software, ensuring that the image interpretation process is operator-independent.

Nevertheless, data concerning the diagnostic validity of OST are still scarce. Most of the performed studies have examined the diagnostic performance of OST, compared with clinical and/or joint US examinations [15, 16, 27]. To date, MRI has been compared with OST in only one study, which was performed during internal validation of the commercial device HandScan^®^ in a small group of rheumatoid arthritis (RA) patients [14]. That constitutes a significant literature gap, since joint MRI is the method with the highest known sensitivity and specificity in the assessment of inflammatory joint activity [28].

Therefore, we sought to investigate the diagnostic performance of OST by examining its correlations with MRI. Additionally, we evaluated associations of OST with clinical and laboratory activity markers as well as patient-related and disease-related characteristics.

## Methods

We analyzed data from *71* consecutive adult patients with a rheumatological diagnosis and who underwent hand-MRI and OST for suspected inflammatory activity, clinically defined as tenderness and/or synovial swelling of at least one finger and/or wrist joint while presenting in a tertiary rheumatology clinic. Patients with a RAMRIS synovitis score of at least 1 in one joint were considered (MRI+), while patients without any joint synovitis (MRI-) served as an intrinsic control group. This classification criterion for MRI positivity was based on the OMERACT definition, which describes synovitis as an area within the synovial compartment exhibiting abnormal post-contrast enhancement, with a thickness greater than that of the normal synovium. This definition takes into account both the contrast enhancement and the thickening of the synovium and a synovitis score of “1’’ indicates mild inflammation in the joint’s synovial compartment [29, 30].

In addition, retrospective data from healthy controls who had undergone OST examinations were taken into account as external validation group [15].

Exclusion criteria included presence of joint prostheses/implants, severe hand deformities like extremely pronounced ulnar deviation or severely mutilated fingers, recent trauma or surgery, a change in immunosuppressant therapy (glucocorticoids and/or DMARDs) between the examinations by MRI and OST, and a time interval exceeding 7 days between OST and MRI scans.

Informed consent was obtained from patients and HC, and the assessment has been approved by the ethics committee of the Rhineland Palatinate State Medical Council, Germany (EC number: 13042).

### Data collection

In all groups, epidemiological and anthropometric data along with other health-related conditions were documented. In both patient groups, clinical examinations were performed and counts of tender (TJC) and swollen (SJC) joints were documented. Moreover, disease activity was assessed on a visual analogue scale (VAS) and laboratory inflammation markers (CRP, ESR) were assessed. Subsequently, clinical disease activity scores (DAS28-ESR, DAS28-CRP) were calculated. Other patient-associated characteristics like current medication, disease-associated antibodies like rheumatoid factor (RF), anti-cyclic citrullinated peptide antibodies (ACPA), and antinuclear antibodies (ANA) were reported. Radiographs of the hands in 2 planes were examined by an experienced radiology specialist to control for the presence of typical RA erosions (marginal), osteophytes, and calcium pyrophosphate dihydrate crystal depositions (chondrocalcinosis).

### Optical spectral transmission

The OST examinations were carried out by trained nursing staff, blinded to the results of the clinical examinations, laboratory values, and MRI findings. At the beginning of the OST examination, participants were asked to put their forearms into the HandScan device through 2 frontal openings that held pressure cuffs. Subsequently, the forearms were positioned on a glass handrest. Red and near-infrared laser light with wavelengths of 660 nm and 808 nm then illuminated the palm-side forearm surface, including wrists, MCP (middle joints), PIP (middle finger joints), and reference areas for each joint. The light transmitted through the hands was recorded by a camera attached to the upper part of the device [14].

The OST measurement was done in approximately 100 s and consisted of three phases: (a) a low cuff pressure phase, (b) an elevated cuff pressure phase [55 mmHg (= 7.3 kPa)], and (c) a second low cuff pressure phase. In the initial phase, the baseline transmission was assessed. Followed by the second phase, an increased cuff pressure-induced blood pooling in the examined areas. During the third phase, cuff pressure decreased, resulting in inversion of venous occlusion and blood pooling.

An integrated software automatically identified “regions of interest” (ROI), including wrists, MCP I-V, and PIP I-V on both sides, along with reference areas positioned distally to the examined joints. Utilizing a comparison between blood flow in the ROI and reference areas, the system functioned as a control mechanism to detect any abnormalities or variations in peripheral blood flow attributed to systemic factors, such as diabetes mellitus, nicotine use, arterial hypertonia or vasoactive medication.

### Magnetic resonance imaging

MRI was performed by radiology staff using a *1.5* Tesla MRI machine (Siemens MAGNETOM Avanto). The MRI Protocol, applied on one hand, consisted of the following sequences: [1] Coronal T1-weighted turbo spin-echo images (field of view (FOV), *23* cm; slice thickness, *1.5* mm) [2], coronal Proton density-weighted images (PD) (FOV, *23* cm; slice thickness, *1.5* mm) [3], transverse PD images (slice thickness, *3* mm) [4], coronal T1-weighted turbo spin-echo images with fat saturation (FS) after the application of contrast agents (FOV, *23* cm; slice thickness, *1.5* mm) [5], transverse T1-weighted turbo spin-echo images with FS and contrast agents (slice thickness, *2.5* mm) and [6] t2-weighted two-dimensional multi-echo Images (Med2D) (slice thickness, *2.5* mm).

**Fig. 1:**
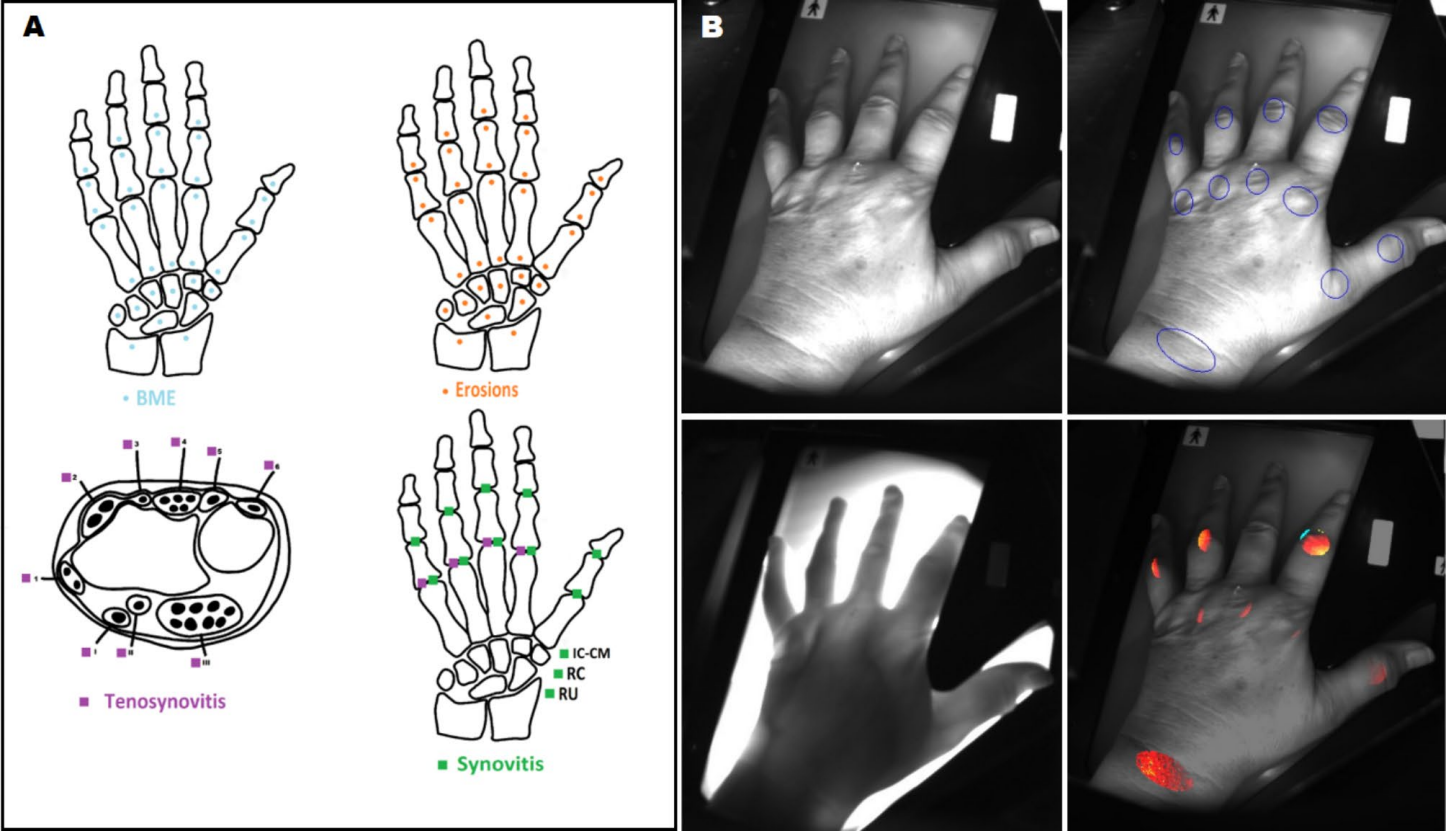
(***A***) Pathologies and regions scored according to the RAMRIS-system. The sketch shows the examined areas for bone marrow edema (top left), bone erosions (top right), and tenosynovitis and synovitis (bottom left and right, respectively) in the wrist and metacarpophalangeal joints. (***B***) Assessment of the inflammation by optical spectral transmission (OST) in wrist and finger joints. 11 joints of each hand were separately examined within each ROI and given an individual score, as well as a cumulative score of the hand. *RAMRIS*: Rheumatoid Arthritis MRI Score; *IC-CM*: intercarpal-carpometacarpal joints; *RC*: radiocarpal joint; *RU*: distal radioulnar joint; *BME*: bone marrow edema; *1–6* and *I-III*: extensor and flexor tendon compartments of the wrist. *The sketch was drawn with affinity designer 2*

These sequences were in accordance with the RAMRIS scoring methodology published in 2003 and updated in 2017 by the MRI OMERACT group [29–33]. Therefore, the scoring system was utilized to assess four typical arthritis findings, such as synovitis, tenosynovitis, bone marrow edema, and erosions, in diverse joint locations as illustrated in (Fig. 1). According to the provided image atlases by OMERACT [34, 35], scoring of MRI findings was performed by a trained physician, who was blinded to the results of other study measurements.

### Scoring of OST and MRI examinations

**Fig. 2:**
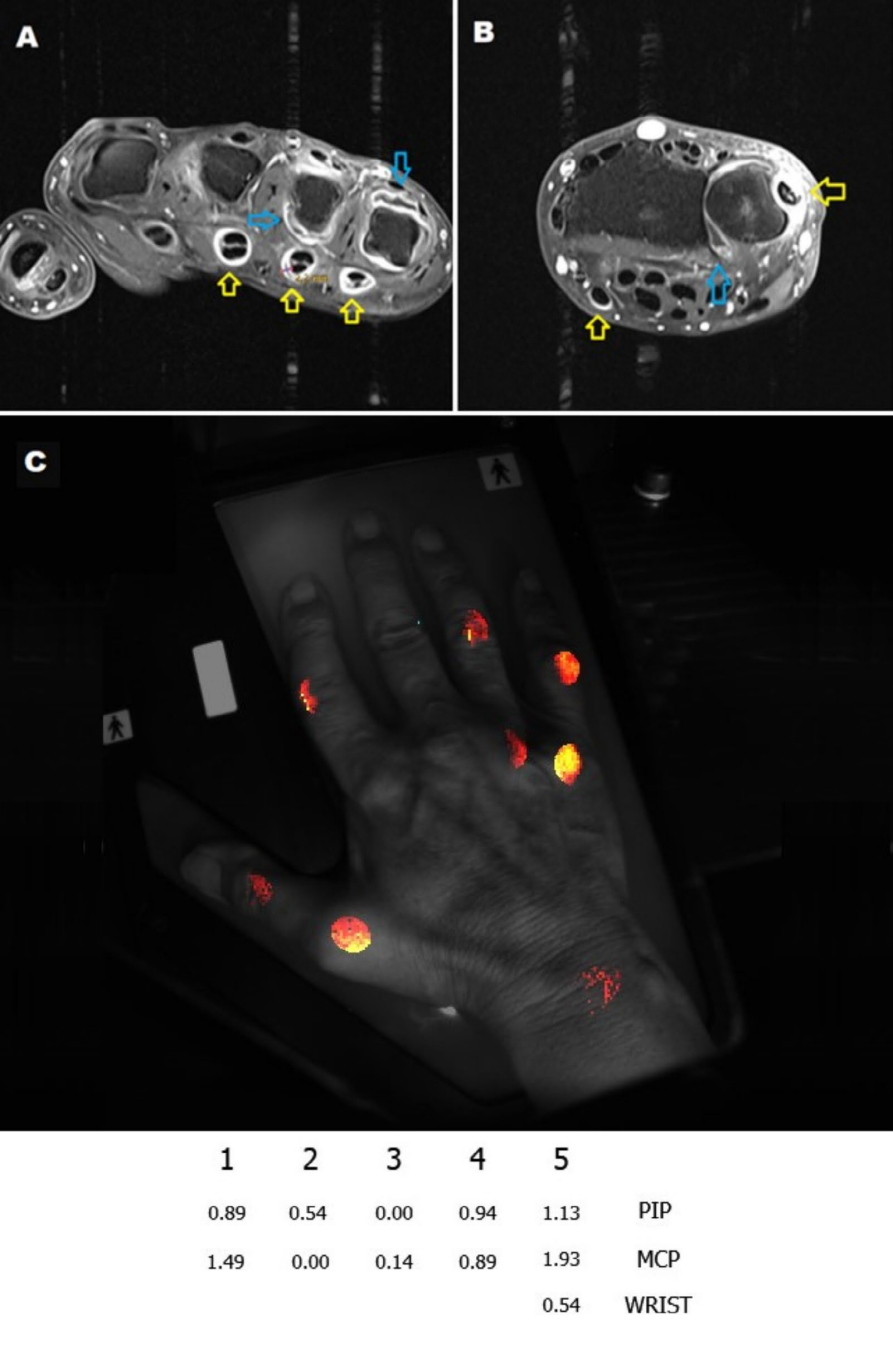
MRI findings of RA Patient and scoring according to RAMRIS in comparison to OST results. (***A***,*** B***) T1-images show hyperintensity in synovial membrane of wrist and MCP joints with hypointense fluid effusion, indicating synovitis (*blue arrows*). RAMRIS synovitis scores of 3/3/1 were assigned to MCP4/5 and radioulnar wrist joint, respectively. Hyperintense signals are observed within the tendon compartments indicating tenosynovitis (*yellow arrows*). The distance between tendon and synovial membrane in MCP4 measured 2.2 mm and scored 2/3. (***C***) OST results of same hand showing individual scores of the wrist, MCP and PIP joints. *RAMRIS*: Rheumatoid Arthritis Magnetic Resonance Imaging scoring, OST: optical spectral transmission

The HandScan device, developed by Demcon-Hemics in the Netherlands, utilizes the OST to measure an individual score for each wrist, metacarpophalangeal (MCP), and proximal interphalangeal (PIP) joint, as well as a cumulative OST score of both hands and each hand separately. In MRI, each joint or bone was also assessed separately according to the RAMRIS-System (Fig. 2). The MRI scores for each specific pathology (synovitis, tenosynovitis, bone marrow edema, and bone erosions) were then summed to calculate a RAMRIS score of one hand for that pathology. The MRI pathology scores were compared to their respective OST score of the same hand. Moreover, individual scores for each pathology of the same joint groups (wrist, MCP, PIP) were then compared to their respective individual OST joint scores of the same hand. Additional details on the scoring methods for OST and MRI are found in the additional file 1.

### Statistical analysis

We evaluated the normality of distribution using the Shapiro-Wilk test and graphical methods like quantile-quantile plots. Additionally, Fisher’s exact test was employed to compare categorical variables among arthritis patients with and without MRI inflammation, and controls. Differences in non-normally distributed metric patient characteristics were assessed using the Mann-Whitney test. Correlations between OST, MRI, and other characteristics were examined using Spearman’s correlation coefficient, while Pearson’s coefficient was used for normally distributed values. Further, we measured the agreement between MRI (RAMRIS synovitis) and OST in each joint category (wrist, MCP and PIP), as well as cumulatively for all joints via Cohen’s kappa. Finally, we applied the independent samples t-test to explore differences in the normally distributed OST values in relation to binary categorical variables for MRI + patients and the RA subgroup.

To ensure an accurate assessment of OST differences between IA patients and control subjects, we have applied a binary logistic regression adjustment model including systemic factors that could influence blood flow like diabetes mellitus, arterial hypertension, and nicotine consumption as well as other patient-specific parameters like sex, age and BMI.

To evaluate the diagnostic performance of OST, ROC analyses were conducted at both the participant and the joint levels. At the patient level, comparisons included “MRI + vs healthy controls” and the subgroup of “RA-diagnosed MRI + patients versus healthy controls” with subsequent assessment of OST-cut-off values. At the joint level, the analysis involved comparing the ordinal RAMRIS synovitis values to the numerical OST values for the wrist, MCP, PIP, and all joints collectively. Additional ROC curves were performed for the comparisons OST vs. RAMRIS tenosynovitis, as well as OST vs. DAS28. A significance level of 0.05 was considered, and all statistical computations were carried out using SPSS software version 27.0.

## Results

Descriptive statistics of all subjects included in the analysis are presented in Table 1.

**Table 1:** Descriptive characteristics by each study group

	MRI+, *n* = 51	Controls, *n* = 114	Significance MRI + vs. controls	MRI-, *n* = 20	Significance MRI + vs. MRI-
OST#	16.41 ± 5.53	10.78 ± 4.19	< 0.001***	11.52 ± 5.03	< 0.001***
age, yrs,†	62.00 (73.00–52.00)	51.00 (57.00–35.00)	< 0.001***	55.50 (63.00-45.25)	0.050
sex (female), %	58.80	77.20	0.010*	80.00	0.080
nicotine use, %	23.40	19.40	0.360	11.10	0.230
arterial HTN, %	53.20	19.40	< 0.001***	44.40	0.360
diabetes, %	19.10	1.90	< 0.001***	11.10	0.360
BMI†	28.10 (32.45–25.73)	25.49 (28.55–21.71)	< 0.001***	26.06 (37.37–23.99)	0.370
RF-positive, %	26.70	-	-	0.00	0.010*
anti-CCP–positive, %	25.50	-	-	0.00	0.020*
tender joint count†	3.00 (10.00–0.00)	-	-	3.00 (10.00–1.00)	0.420
swollen joint count†	1.00 (3.00–0.00)	-	-	0.00 (2.00–0.00)	0.370
VAS, mm†	50.00 (70.00–30.00)	-	-	40.00 (70.00–40.00)	0.820
DAS28-ESR#	4.13 ± 1.51	-	-	4.13 ± 0.74	0.540
DAS28-CRP#	3.77 ± 1.31	-	-	3.55 ± 0.66	0.180
disease duration, yrs, †	2.15 (6.55 − 0.46)	-	-	0.03 (1.38 − 0.02)	0.010*
erosions, %	22.50	-	-	6.70	0.170
osteoarthritis, % §	49.00	-	-	50.00	0.430
NSAID, %	25.50	-	-	22.20	0.530
immunosuppressants, %	38.30	-	-	16.70	0.080
csDMARD, %	27.70	-	-	11.10	0.140
bDMARD, %	12.80	-	-	5.60	0.370
JAK inhibition, %	0.00	-	-	0.00	-
glucocorticoids (low dose), %	57.40	-	-	29.40	0.040*
no DMARD, %	61.70	-	-	83.30	0.070

*p < 0.05, **p < 0.01, ***p < 0.001. #Data are presented as mean ± standard deviation, as they are normally distributed. †Data are presented as median (IQR), as they are not normally distributed. §Distal interphalangeal joints excluded because they were not examined by OST or MRI. *OST*: optical spectral transmission; *HTN*: hypertension; *BMI*: body mass index; *RF*: rheumatoid factor; *anti-CCP*: anticyclic citrullinated peptide antibodies; *VAS*: visual analog scale; *DAS28-ESR*: 28-joint count Disease Activity Score based on erythrocyte sedimentation rate; *DAS28-CRP*: 28-joint count Disease Activity Score based on C-reactive protein; *NSAID*: non-steroidal anti-inflammatory drug; c*sDMARD*: conventional synthetic disease-modifying antirheumatic drugs; *bDMARD*: biologic DMARD; *JAK*: Janus kinase.

Overall, *51* of the *71* patients [RA (n = *31*), psoriatic arthritis (PsA) (*n* = 13), gout (*n* = 2), mixed connective tissue disease (*n* = 2), and undifferentiated arthritis (*n* = 3) [36–39]] (*1*,*104* wrist and finger joints in total) were MRI+. A total of *20* patients were included in the intrinsic control/MRI- group [hand osteoarthritis (*n* = 10), fibromyalgia syndrome (*n* = 6), systemic lupus erythematosus (*n* = 1), systemic sclerosis (*n* = 1), HLA-B27 associated arthritis (*n* = 1), and non-specific non-inflammatory arthralgia (*n* = 1)] (*438* wrist and finger joints in total). The HC group consisted of *114* participants (*2*,*508* joints in total).

A total of 20 joints were excluded by the OST software, while a total of 94 joints were excluded from the MRI due to them not being fully captured by the scan or having metal artifacts. OST measurements were performed in the same joints as MRI (*1*,*104* joints of *51* patients with MRI+, *438* joints of *20* MRI- patients and in *2*,*508* joints of *114* of HC).

### Correlations between MRI and OST

Among MRI + patients, statistical correlation analyses revealed significant correlations between the OST score and the RAMRIS synovitis score of the hand (*rho = 0.541;* *p* < 0.001,* κ = 0.330;* *p* < 0.001), along with a significant but moderate correlation with the RAMRIS-tenosynovitis of the hand at the wrist and MCP level combined (*rho = 0.341;* *p* = 0.010). However, there was no direct significant correlation between MRI-detected synovitis and tenosynovitis scores (*rho = 0.261;* *p* = 0.124).

At the joint level, OST correlated strongly with the RAMRIS synovitis score of the wrist (*rho = 0.644;* *p* < 0.001,* κ = 0.284; p* < 0.027), MCP (*rho = 0.582; p* < 0.001,* κ = 0.287;* *p* < 0.001), and PIP-joints (*rho = 0.566;* *p* < 0.001,* κ = 0.402;* *p* < 0.001), respectively. Additionally, there was a significant but moderate correlation between the OST-MCP score and the RAMRIS-tenosynovitis score at the level of MCP-joints (*rho = 0.351; p* < 0.008) (Table 2).

**Table 2:** Associations of OST and RAMRIS scores in MRI + and RA diagnosed MRI + patient groups

variable 1	variable 2	Spearmann’s rho	significance (*p*) (2-sided)
**MRI + patients**,***n* = 51**			
OST hand	RAMRIS-synovitis hand	0.541	< 0.001***
OST MCP	RAMRIS-synovitis MCP	0.582	< 0.001***
OST PIP	RAMRIS-synovitis PIP	0.566	< 0.001***
OST wrist	RAMRIS-synovitis wrist	0.644	< 0.001***
OST hand	RAMRIS-tenosynovitis	0.341	0.010*
OST MCP	RAMRIS-tenosynovitis MCP	0.351	0.008**
OST wrist	RAMRIS-tenosynovitis wrist	-0.036	0.402
OST hand	RAMRIS-BME hand	0.201	0.106
**RA diagnosed MRI+**,***n* = 31**			
OST hand	RAMRIS-synovitis hand	0.604	< 0.001***
OST MCP	RAMRIS-synovitis MCP	0.753	< 0.001***
OST PIP	RAMRIS-synovitis PIP	0.589	< 0.001***
OST wrist	RAMRIS-synovitis wrist	0.714	< 0.001***
OST hand	RAMRIS-tenosynovitis	0.490	0.003**
OST MCP	RAMRIS-tenosynovitis MCP	0.387	0.017*
OST wrist	RAMRIS-tenosynovitis wrist	0.096	0.304
OST hand	RAMRIS-BME hand	0.158	0.220

*p < 0.05, **p < 0.01, ***p < 0.001

Spearman’s correlation tests were performed to investigate the relationships between OST and MRI (rho: spearman’s correlation coefficiency). *OST*: optical spectral transmission; *RAMRIS*: Rheumatoid Arthritis Magnetic Resonance Imaging Score; *BME*: bone marrow edema.

The comparison with the RA-diagnosed MRI + patients demonstrated similarly strong correlations between the OST total hand score, the RAMRIS synovitis hand score *(rho = 0.604*, *p* < 0.001,* κ = 0.390; p* < 0.001), and the RAMRIS-tenosynovitis hand score *(rho = 0.490*, *p* = 0.003). Correlations of OST and RAMRIS synovitis score were also found to be strong within the separate individual joint categories. The strongest correlation was measured at the MCP-joints (*rho = 0.753;* *p* < 0.001,* κ = 0.366;* *p* < 0.001), followed by the wrist- (*rho = 0.714;* *p* < 0.001, *κ = 0.374;* *p* < 0.020), and PIP-joints (*rho = 0.589;* *p* < 0.001,* κ = 0.494;* *p* < 0.001), respectively. At the MCP joint level, a moderate correlation was observed between the OST-MCP score and RAMRIS-tenosynovitis score (*rho = 0.387;* *p* < 0.017).

### Receiver operating characteristics

**Fig. 3:**
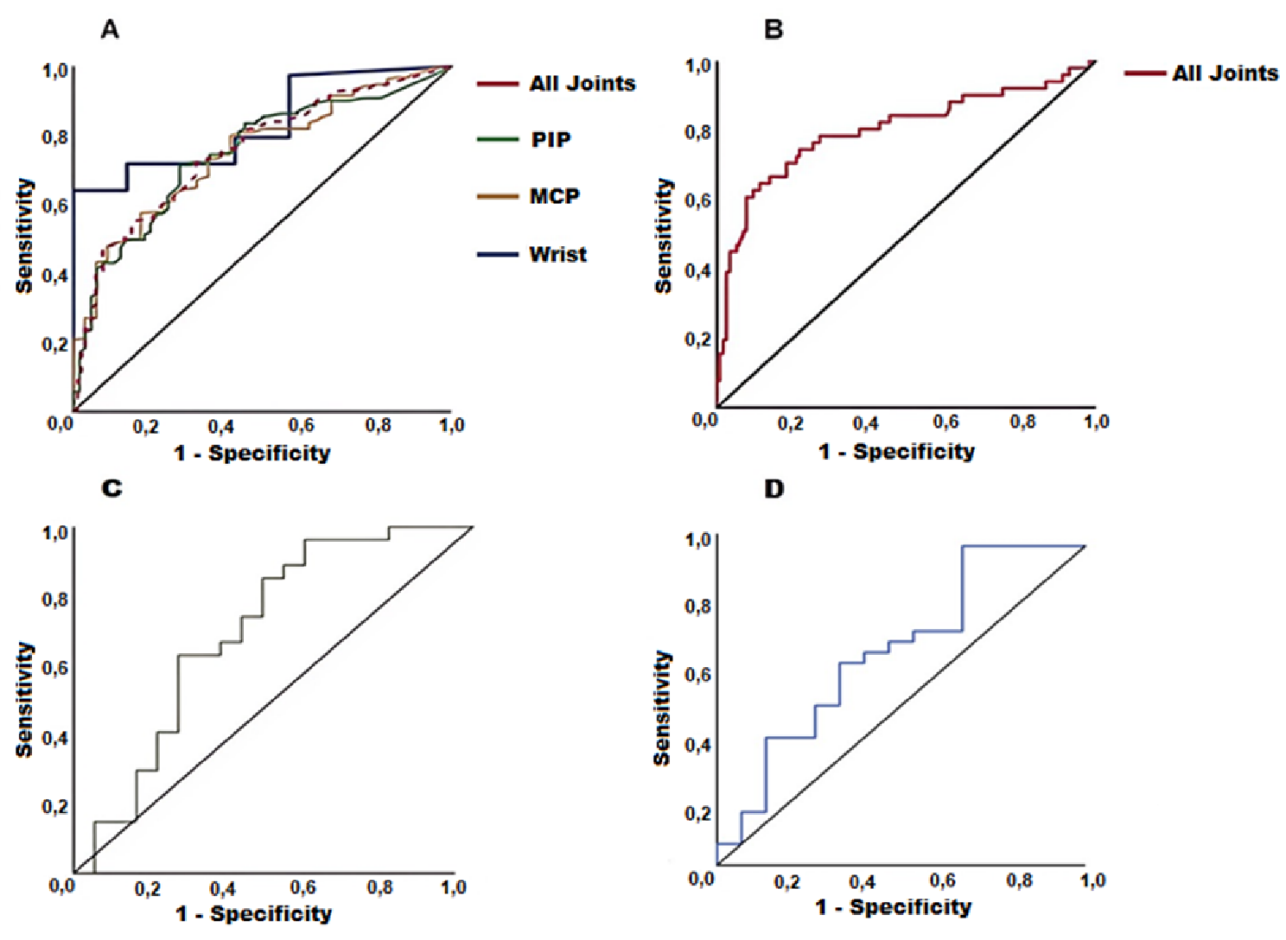
Receiver-operating characteristic (ROC) curves and OST values of patients with IA (MRI+). (***A***) ROC between OST and RAMRIS synovitis score (reference) at the joint level: OST area under the curve for wrist joints (AUC 0.833; 95% CI 0.700–0.966), metacarpophalangeal joints (AUC 0.723; 95% CI 0.634–0.812), proximal interphalangeal joints (AUC 0.750; 95% CI 0.680–0.820), and all joints combined 0.755 (95% CI 0.706–0.805). (***B***) ROC between inflammatory arthritis and healthy controls at the patient level (all joints): AUC = 0.800 (95% CI 0.715–0.882), (***C***) ROC between OST and RAMRIS tenosynovitis score: AUC = 0.698 (95% CI 0.533–0.863), (***D***) ROC between OST and DAS28 score: AUC = 0.670 (95% CI 0.491–0.842). *OST*: optical spectral transmission, *RAMRIS*: Rheumatoid Arthritis Magnetic Resonance Imaging scoring

ROC curves were employed for the comparisons of the “MRI + cohort vs. healthy control group” and the “RA MRI + subgroup vs. healthy control group”. The ROC for “MRI + cohort vs healthy controls” showed an area under the curve (AUC) of *0.800* (*95% CI 0.715–0.882*), with a sensitivity of *0.784* and specificity of *0.728* for an OST cutoff of *13.28* (Youden index *0.512*, positive likelihood ratio LR + *2.882*, LR– *0.296;* Fig. 3), while the ROC for “RA MRI + subgroup vs. healthy controls” revealed an AUC of *0.791* (95% CI *0.679–0.902*) with a sensitivity of *0.806* and specificity of *0.728* for a cut-off OST value of *13.37* (Youden index *0.534*, positive likelihood ratio LR + *2.963*, LR– *0.266*).

ROC analyses were also conducted to assess the diagnostic performance of OST in comparison to MRI. A joint was considered inflamed when the RAMRIS synovitis score was ≥ 1 [29, 30]. The inflammation status identified by MRI for joints of the same joint group was then compared with its respective OST score of the same joint group. The analysis was, therefore, performed separately for three joint categories: MCP, PIP, and wrist joints. The overall AUC for all joints was *0.755* (*95% CI 0.706–0.805*). The highest diagnostic performance was observed at the wrist joints (*AUC 0.833; 95% CI 0.700–0.966*), followed by the PIP (*AUC 0.750; 95% CI 0.680–0.820*) and MCP joints (*AUC 0.723; 95% CI 0.634–0.812*).

ROC analyses were also performed to evaluate the relationship between OST and the RAMRIS tenosynovitis score. In the IA (MRI+) cohort, the ROC analysis revealed an AUC of *0.698 (95% CI 0.533–0.863*, Fig. 3), while in RA diagnosed MRI + group an AUC of *0.766 (95% CI 0.552–0.980)*.

Finally, ROC analyses comparing OST with DAS28 were conducted. In the IA (MRI+) group, ROC showed an AUC of *0.670 (95% CI 0.491–0.842*, Fig. 3), whereas in RA diagnosed MRI + group, AUC had a value of *0.758 (95% CI 0.509–0.977).*

### OST differences between patients and healthy controls

Mean OST values were significantly higher in the MRI + patient group compared to the MRI- patient group (*16.41* ± *5.53 vs. 11*,*52 ± 5.03*, p < *0.001*), and to HC (*16.41 ± 5.53 vs. 10.78 ± 4.19*, p < *0.001*), respectively. Even after the statistical adjusting analysis for possible confounding factors via multivariate binary logistic model, OST remained statistically significant in the IA patient group compared to the control group (*0.829*,* 95% CI 0.740–0.929; p*_*adj*_*= 0.001*) (additional file 1). This suggests that individuals with MRI + inflammatory arthritis exhibit higher OST values than those in the HC group.

### Correlations of OST within the patient groups

Among MRI + patients, statistical correlation analyses showed moderately significant associations between OST and TJC (rho *= 0.333*; p = *0.013*), SJC (*rho 0.308;* *p* = 0.017), age (*rho 0.300;* *p* = 0.016), DAS28-CRP (*r* = 0.312; *p = 0.018*), and weaker, yet significant correlations with DAS28-ESR (*r* = 0.297; *p = 0.024*). Among the RA subgroup, there was a stronger significant correlation between OST and SJC (*rho = 0.486; p* = 0.004), TJC (*rho = 0.372;* *p* = 0.031), DAS28-ESR (*r* = 0.426; *p = 0.015*) and DAS28-CRP (*r* = 0.506; *p*** = 0.004**). No significant correlations were observed between OST and other factors such as BMI, visual analogue scale, gender, nicotine use, arterial hypertension, diabetes mellitus, erosions in conventional radiographs, osteoarthritis, RF–/ACPA positivity and immunosuppressants or glucocorticoids intake (all *p* > 0.05; Table 3).

**Table 3:** Correlations between OST and patients’ characteristics in MRI + and RA diagnosed MRI + patient groups

metrical variables	correlation coefficiency (Spearmann’s rho/ Pearson’s *r*)	significance (*p*)	correlation coefficiency (Spearmann’s rho/ Pearson’s *r*)	significance (*p*)
	**MRI+**,***n* = 51**		**RA diagnosed MRI+**,***n*** = 31	
age, yrs†	rho = 0.300	0.016*	rho = 0.269	0.072
BMI, kg/m†	rho=-0.233	0.066	rho=-0.284	0.072
TJC†	rho = 0.333	0.013*	rho = 0.372	0.031*
SJC†	rho = 0.308	0.017*	rho = 0.486	0.004**
VAS, mm†	rho=-0.151	0.155	rho=-0.019	0.462
DAS28-ESR#	*r* = 0.297	0.024*	*r* = 0.426	0.015*
DAS28-CRP#	*r* = 0.312	0.018*	*r* = 0.506	0.004**
categorial variables	mean OST score ± SD	significance (p)	mean OST score ± SD	significance (p)
	**MRI+**,***n* = 51**		**RA diagnosed MRI+**,***n* = 31**	
sex: female	15.23 ± 5.71	*P* = 0.059	16.13 ± 6.28	0.587
male	18.10 ± 4.91		17.39 ± 5.78	
nicotine use: no	16.57 ± 5.73	*P* = 0.944	15.93 ± 6.75	0.406
yes	16.44 ± 5.39		18.03 ± 5.17	
hypertension: no	17.21 ± 5.59	*P* = 0.451	17.68 ± 6.69	0.430
yes	15.96 ± 5.66		15.66 ± 6.23	
diabetes: no	16.11 ± 5.76	*P* = 0.237	16.28 ± 6.44	0.758
yes	18.37 ± 4.71		17.47 ± 6.71	
erosions: no	14.90 ± 5.48	*P* = 0.160	14.14 ± 5.75	0.370
yes	17.01 ± 4.80		16.88 ± 5.84	
osteoarthritis: no §	16.78 ± 6.49	*P* = 0.393	15.93 ± 7.58	0.898
yes	15.24 ± 4.71		15.59 ± 5.33	
RF: negative	15.89 ± 5.56	*P* = 0.247	15.01 ± 6.40	0.149
positive	18.25 ± 5.95		19.03 ± 6.45	
anti-CCP: negative	15.87 ± 5.53	*P* = 0.171	15.10 ± 6.50	0.159
positive	18.51 ± 5.56		18.54 ± 5.83	
immunosuppressants: no	16.45 ± 5.72	*P* = 0.892	16.01 ± 6.59	0.698
yes	16.68 ± 5.55		16.97 ± 6.32	
glucocorticoids: no	16.97 ± 6.35	*P* = 0.670	16.89 ± 7.51	0.806
yes	16.23 ± 5.07		16.21 ± 5.86	

*p < 0.05, **p < 0.01, ***p < 0.001. Both Spearman and Pearson correlation test were conducted to examine the associations between OST score and the metrical variables. # Pearson correlation tests were performed since the data is normally distributed (r: Pearson correlation index). †Spearman correlation tests were performed as the data is not normally distributed (rho: Spearman correlation index). OST scores were normally distributed in the MRI + and RA diagnosed MRI + patient groups. t-test was used to investigate the relationships between OST and categorial patient characteristics. § Distal interphalangeal joints excluded because they were not examined by OST or MRI. *OST*: optical spectral transmission; *BMI*: body mass index; *TJC*: tender joint count; *SJC*: swollen joint count; *VAS*: visual analog scale; *DAS28-ESR*: 28-joint count Disease Activity Score based on erythrocyte sedimentation rate; *DAS28-CRP*: 28-joint count Disease Activity Score based on C-reactive protein, *RF*: rheumatoid factor; *anti-CCP*: anticyclic citrullinated peptide antibodies

## Discussion

In this study, we compared the performance of OST with MRI findings in patients with and without inflammatory arthritis. Our data indicate that OST performs similarly to MRI in detecting inflammatory activity in patients with inflammatory arthritis and positive findings on hands MRI. We observed significant correlations between OST and MRI activity scores independent of clinical diagnosis for arthritis across all joints but also at individual joint areas, such as wrist, metacarpophalangeal, and proximal interphalangeal joint levels.

To our knowledge, this is the largest exploration to examine associations of OST with MRI findings among patients with inflammatory arthritis. We are aware of only one further study, performed in the context of internal validation, to ever compare OST with MRI in a small group of RA patients [14]. In this study, the RAMRIS system was also utilized for scoring synovitis and bone marrow edema. However, MRI scans were limited to RA patients in remission or with low disease activity, focusing exclusively on the wrist and MCP joints and combining both to generate a total RAMRIS synovitis score. Moreover, there were no examinations of tenosynovitis and erosions. In our investigation, we conducted a novel analysis by separately assessing OST joint scores alongside MRI scores for wrists, MCP, and PIP joints, and collectively across all joints. Our focus encompassed four pathologies that are associated with inflammatory arthritis: synovitis, tenosynovitis, bone marrow edema, and bone erosions. ROCs between OST and RAMRIS-synovitis as a reference, showed the best OST performance at wrist joint level, followed by PIP and MCP. Interestingly, the first studies on OST which have used the primary HandScan device had indicated a worse diagnostic performance of OST at the wrist level compared to MCP and PIP joints [14, 24]. However, in both the present and previous studies of our group [15, 16], the most modern HandScan version with an installed new light source was used, which has been found to be associated with improved diagnostic performance at the wrist level also by further research groups [24].

Interestingly, van Onna et al. found that OST correlated significantly with the combined RAMRIS-synovitis score of the wrist and MCP joints of one hand [14]. PIP joints were however not included in the MRI scoring. In our study, we were able to show significant correlations of RAMRIS synovitis with OST for all 3 examined joint categories (wrist, MCP, and PIP), as well as for the whole hand.

In our study, OST was found to correlate with DAS28 scores in RA patients. However, OST correlations with DAS28-CRP were stronger than with DAS28-ESR. One possible explanation for this finding is a confounding effect of factors unrelated to disease activity on ESR like age [40], anemia [41], or hypergammaglobulinaemia [42]. Moreover, OST correlated stronger with SJC than with TJC in RA patients. Other studies have also shown a stronger OST correlation with SJC than TJC [14, 15, 43], supporting its potency to detect the inflammatory nature of arthralgia.

The results of the present study are important, given the necessity for reliable assessment tools for individuals with inflammatory arthritis. Indeed, joint MRI should be further considered the gold standard for diagnosing inflammatory arthritis in routine clinical practice, next to joint ultrasound, given its numerous advantages, particularly its diagnostic accuracy. Unlike conventional imaging techniques, MRI offers higher sensitivity in detecting early inflammatory changes, synovitis, bone marrow edema, and erosions, even in cases of subclinical disease courses [44, 45]. On the other hand, OST has demonstrated acceptable discriminative performance in patients with inflammatory arthritis in both this study and previous research by our group, making it a promising tool that warrants further investigation [15, 16].

Moreover, OST has some practical advantages over MRI: OST examinations are in general less expensive than MRI, can be performed bilaterally, and do not require the use of contrast agents [46]. Additionally, OST software provides an examiner-independent joint inflammatory activity score for each patient, offering objective scoring that may surpass the clinical examination. An OST assessment, which examines all wrist and finger joints of both hands simultaneously, also takes significantly less time than a clinical examination of each individual joint. Therefore, we believe OST can be considered a complementary tool to MRI and other established diagnostic methods.

Consistent with the results reported by van Onna, et al., we were not able to find significant correlations between OST and the RAMRIS bone marrow edema score [14]. This observation is reasonable given that red/infrared light, utilized in OST, primarily detects synovial inflammatory changes and is less effective in identifying bone pathologies due to its limited ability to penetrate bony structures.

Our study has some limitations. To begin with, this is a retrospective exploration which could be subject to selection bias. However, identification of the included patients has been performed upon clinical indication during inpatient stays in a real-life medical setting, independently of this study. Moreover, we have recruited all patients who have received MRI and OST due to suspected inflammatory activity in a consecutive manner.

Further, to avoid statistical errors that could arise from the inclusion of different inflammatory arthritis types we have focused on the examination of the whole arthritis group and only one subgroup including solely RA patients. Further subgroup analyses for other types of inflammatory arthritis with low patient counts were not performed.

An additional limitation comes from the fact that we have focused on inflammatory arthritis patients who were MRI-positive. Thus, the diagnostic utility of OST in MRI-negative inflammatory arthritis patients could not be investigated in the context of the present exploration. However, in a previous study of our group, the diagnostic performance of OST was also assessed in a RA patient cohort with various grades of disease activity (including patients in remission) and has been found to be acceptable [15].

Relevant conclusions regarding the relationship between RAMRIS erosion score and OST could not be drawn due to the small number of patients with typical (marginal) erosions in this cohort. Interestingly, we found a significant correlation between the OST score and the RAMRIS tenosynovitis score of the hand particularly at the level of the MCP joints. This correlation seems to be independent of synovitis, since no significant correlations between RAMRIS synovitis and tenosynovitis scores were detected. However, when examining the wrist, we observed no significant correlations between the OST wrist score and its corresponding RAMRIS tenosynovitis wrist score. In general, the OST device is not supposed to provide tendon-specific scores, since it is primarily conceived for the quantitative assessment of synovitis in the wrist, MCP, and PIP joints, and not for their adjacent tendons.

Moreover, OST can detect inflammation only in the wrist, MCP, and PIP. Therefore, the inflammatory status of the DIP joints could not be examined and was deliberately not considered in MRI scoring. Despite this limitation, we were able to show positive proof of principle regarding the validity of OST in examining inflammation of all included joint groups. Finally, OST primarily measures light absorption in blood flow in the joints, making it less anatomically specific, compared to joint ultrasound and MRI. In cases of severe finger deformities like extremely pronounced ulnar deviation or severely mutilated fingers, the OST software may have difficulties in locating these anatomically abnormal joints and thus include only a part of the joints in the final calculation. For that reason, these cases has been excluded from our study following the methodology by other researchers on this topic [24].

## Conclusion

Our findings indicate significant associations between OST scores and MRI activity scores for synovitis and tenosynovitis. However, no significant correlation was observed between OST and MRI score for bone marrow edema. Moreover, we were able to show strong correlations of OST with clinical activity parameters, like SJC and DAS28, and demonstrated excellent performance in differentiating IA patients from controls. OST may present a promising diagnostic tool, due to its non-invasive nature and lower resource requirements. Control and confirmation of these results in future studies are warranted.

## Electronic supplementary material

Below is the link to the electronic supplementary material.

Supplementary Material 1

## Data Availability

The data analysed during the current study are available from the corresponding author on reasonable request.

## Abbreviations

OST: Optical spectral transmission
MRI: Magnetic resonance imaging
IA: Inflammatory arthritis
US: Ultrasound
RAMRIS: Rheumatoid arthritis magnetic resonance imaging score
MRI + Patient: Group with inflammatory signs on MRI examinations
MRI-: Patient group with no inflammatory signs on MRI examinations
AUC: Area under the curve
RA: Rheumatoid arthritis
PsA: Psoriatic arthritis
DAS28-ESR: Disease activity score calculated on 28 joints based on erythrocyte sedimentation rate
DAS28-CRP: Disease activity score calculated on 28 joints based on c-reactive protein
RF: Rheumatoid factor
ACPA: Anti-cyclic citrullinated peptide antibodies
ANA: Antinuclear antibodies
FOI: Fluorescence optical imaging
MCP: Metacarpophalangeal joints
PIP: Proximal interphalangeal joints
BMI: Body mass index
TJC: Tender joint count
SJC: Swollen joint count
VAS: Visual analog scale
ROI: Regions of interest
FOV: Field of view
RD: Proton density
TSE: Turbo spin echo
Med2d: Two-dimensional multi-echo
FS: Fat saturation
ROC: Receiver operating characteristic
HC: Healthy control
