# Supplementary material for: Diagnostic performance of optical spectral transmission compared to magnetic resonance imaging in patients with inflammatory arthritis
Source: Arthritis Res Ther. 2025 Jan 30;27:17. doi: 10.1186/s13075-025-03478-y (PMC11780789; doi:10.1186/s13075-025-03478-y)
Supplement: Supplementary file 1 — Supplementary Material 1 [file 13075_2025_3478_MOESM1_ESM.docx]

# Additional material (1)

## **OST scoring algorithm**

The Hemics HandScan® device allocates an individual score ranging from 0 to 3 for each joint examined (wrist, MCP, PIP). In most cases, the total joint count is set at 22, consisting of 10 MCP, 10 PIP, and 2 wrists of both hands. However, variations may occur due to missing joints resulting from amputation or anatomical abnormalities.

The next step involves dividing the sum of the individual scores, multiplied by the number of all measured joints, by the specific number of joints in a certain group. For instance, to calculate the score for both hands of a person without any missing fingers or abnormalities, the 22 individual scores are added up and multiplied by 22, and the result is then divided by 22. Similarly, for a separate hand score, the sum of individual scores for 11 joints is multiplied by 22 and divided by 11. Hasan, AA, et al. have provided an equation to calculate OST score (18):

Total optical score = Average of OST in the measured joints × 22

This developed algorithm maintains OST scores in the same range regardless of the number of measured joints or if, for example, only one hand measurement is performed, which is technically possible.

## **Scoring of MRI**

The RAMRIS system of OMERACT was used to evaluate the MRI images on aspects of inflammatory arthritis such as synovitis, tenosynovitis, bone marrow oedema and bone erosions (29,30). The RAMRIS scoring has an acceptable inter-rater reliability in the assessment of synovitis, tenosynovitis, bone marrow oedema and erosions, as well as a very good intrareader reliability, good inter-reader reliability and a high degree of sensitivity to change (31-33).

To simplify the RAMRIS scoring process, atlases with reference images to facilitate the assessment of joint pathology in RA according to RAMRIS scoring were also provided by OMERACT. These show scoring examples of MRI images of wrist and MCP joints of RA patients for multiple pathologies such as synovitis, bone swelling and bone erosion (34,35).

In our study, synovitis assessment was performed in different regions of the wrist, including radioulnar, radiocarpal as well as combined intercarpal and carpometacarpal joints. Each region was given an individual score of (0/3), and the overall score for the wrist was calculated by adding up these scores (0/9). A similar assessment was also performed for the MCP joints (MCP1-5) and the PIP joints (PIP1-5), with scores of (0/15) for each joint group. By summing the scores for all wrists, MCP and PIP joints of one hand, a total RAMRIS-synovitis score was determined, which represented a value of (0/39). Synovitis was mainly assessed by comparing coronal t1-weighted sequences with fat saturation (FS) before and after the administration of contrast medium. Attention was paid to an increase in signal intensity in the inflamed areas in which the contrast medium has accumulated. Transversal t1-weighted sequences with (FS) after the administration of contrast medium were also compared with the OMERACT atlas images.

The evaluation of tenosynovitis was performed at two joint levels: at the wrist joint level, 6 tendon compartments of the extensor tendons (1: Abductor pollicis longus and extensor pollicis brevis, 2: Extensor carpi radialis longus and extensor carpi radialis brevis, 3: Extensor pollicis longus, 4: Extensor digitorum communis and extensor digitus proprius, 5: Extensor digiti quiniti proprius, 6: Extensor carpi ulnaris) and 3 tendon compartments of the flexor tendons (I: Flexor carpi radialis, II: Flexor pollicis longus, III: Ulnar bursa, including flexor digitorum superficialis and profundus tendons) were examined, with a score of (0/3) assigned to each compartment. The total scores were (0/18) for the extensor tendons and (0/9) for the flexor tendons, respectively (0/27) for a total wrist tenosynovitis score. In addition, the flexor tendons were assessed at the level of the second to fifth MCP joints, resulting in a total score of (0/12). In our investigation, we examined three sequences on tenosynovitis findings: t2-weighted images, proton density (PD) weighted images with (FS), and t1-weighted images with (FS) following the application of the contrast agent, with a focus on observing signal enhancement.

Assessment of bone marrow oedema was performed individually for each bone. For the distal radius and ulna, scores of (0/3) were assigned to a depth of 1 cm from the articular surface of the bone. Each carpal bone received a score of (0/3). The proximal portions of the metacarpals (metacarpus 1-5) were considered up to a depth of 1 cm proximal to the bone, and each was given a score of (0/3). These individual scores were added together to obtain a total score of (0/45) for the whole wrist. For the MCP joints, each MCP1-5 was divided into two parts, with scores of (0/3) assigned to each part - the metacarpal head (proximal) and the phalangeal base (distal). Thus, each MCP received a score of (0/6) and all MCP joints together received a total score of (0/30). The PIP joints were also subdivided into two parts, with each PIP joint having a score of (0/6) and all PIP joints together receiving a total score of (0/30). All individual scores (wrist, MCP, PIP) were summed to obtain a total score of (0/105) for bone marrow oedema of the entire hand. For that purpose, PD-weighted sequences with (FS) were examined for signal enhancement in both the transverse and coronal planes. In addition, after injection of the contrast agent, transversal/coronal t1-weighted sequences with (FS) were checked for contrast agent accumulated in suspected bone marrow oedema areas.

In this study bone erosion was assessed in the exact way to bone marrow oedema. However, scores of (0/10) were assigned for each individual bone. This included the distal radius, distal ulna, each carpal bone, the proximal portions of MC1-5, each section of the MCP and PIP joints (proximal and distal part). This resulted in a total score of (0/150) for the wrist, (0/100) for MCP or PIP joints, and (0/350) for the entire hand. Coronal t1-weighted sequences before and after administration of the contrast agent were considered in the evaluation of bone erosions, as well as the transversal PD and t2-weighted sequences as secondary plane.

**Adjusting for possible confounding effects of OST**

We used a binary logistic regression model to assess the difference of OST between the patient and control group adjusting for factors that could influence systemic blood flow changes like arterial hypertension, diabetes mellitus, and nicotine consumption as well as patient-associated parameters like sex, age and BMI. Even after the statistical adjusting analysis, OST remained statistically significantly higher in the IA patient group compared to the control group as presented in the following table (*p = 0.001*).

|  | Regression coefficient B | Standard error | Wald | df | Sig. (p) | Exp(B) | 95% confidence interval for EXP(B) | |
| --- | --- | --- | --- | --- | --- | --- | --- | --- |
|  |  |  |  |  |  |  | Lower value | Upper value |
| OST | -0.187 | 0.058 | 10.381 | 1 | 0.001 | 0.829 | 0.740 | 0.929 |
| Sex | -0.697 | 0.608 | 1.314 | 1 | 0.252 | 0.498 | 0.151 | 1.640 |
| Age | -0.081 | 0.024 | 11.375 | 1 | 0.001 | 0.922 | 0.880 | 0.967 |
| Body Mass Index | -0.121 | 0.050 | 5.864 | 1 | 0.015 | 0.886 | 0.803 | 0.977 |
| Art. Hypertension | 0.685 | 0.554 | 1.528 | 1 | 0.216 | 1.984 | 0.670 | 5.880 |
| Diabetes mellitus | -0.257 | 1.211 | 0.045 | 1 | 0.832 | 0.773 | 0.072 | 8.295 |
| Nicotine | 0.874 | 0.629 | 1.931 | 1 | 0.165 | 2.395 | 0.699 | 8.212 |
| Constant | 10.330 | 2.904 | 12.652 | 1 | 0.000 | 30652.153 |  |  |
